# Lymphoepithelial pancreatic cyst: A rare entity among pancreatic cystic lesions. Report of two cases

**DOI:** 10.1002/ccr3.2574

**Published:** 2019-12-05

**Authors:** Spyridon Davakis, Athanasios Syllaios, Eleandros Kyros, Nikolaos Dimitrokallis, Stamatios Orfanos, Michail Vailas, Alexandros Papalampros, Evangelos Felekouras

**Affiliations:** ^1^ First Department of Surgery Laiko General Hospital National and Kapodistrian University of Athens Athens Greece

**Keywords:** cystic lesions, Lymphoepithelial cysts, pancreas

## Abstract

Lymphoepithelial pancreatic cysts are extremely rare benign pancreatic cystic lesions. High suspicion and an individual approach are imperative for the best management of those extremely rare entities.

## Graphical abstract

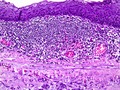

## 1 INTRODUCTION

Lymphoepithelial pancreatic cysts are rare pancreatic cystic lesions. We report two cases of LECs, one patient that was treated with surgical resection and another patient that was managed conservatively. High suspicion and an individual approach are imperative for the best management of those extremely rare entities.

Cystic lesions of the pancreas are well‐known entities that have been recognized in up to 24% of the general population in various autopsy reports.^1^ The increasing use of high‐quality imaging studies has led to an ever‐increasing number of detected cysts of the pancreas. Many studies have been published so far trying to summarize the diagnostic criteria for this entity^2^, ^3^^.^ The clinical history and the histopathologic classification of the cystic lesions of the pancreas vary. They can represent benign, premalignant, or even invasive malignant lesions. Lymphoepithelial cysts (LECs) of the pancreas were first described by Luchtrath and Schriefers in 1985, and approximately 200 have been reported in the English literature so far^4^, ^5^^.^ They represent true pancreatic cysts, and they are characterized histologically by mature squamous epithelium lining, surrounded by multiple lymphocytes or lymphoid follicles filled with keratinizing material.^6^ Although benign in nature, they are clinically difficult to distinguish from malignant lesions such as mucinous cystic neoplasms (MCNs), since patients present with nondiagnostic symptoms, laboratory and imaging examinations.^5^ As a result, patients are usually operated on the suspicion of malignancy and only a few cases have been described to receive conservative treatment.^7^ We report two cases of LEC treated in our department: the first concerning a lesion on the tail of the pancreas that was excised due to suspicion being a tumor of mesenchymal origin but turned out to be a case of LEC of the pancreas, and the second concerning a lesion with a definite diagnosis of LEC of the pancreas that has been managed conservatively by observation with regular imaging studies for the last 5 years.

## 2 CASE PRESENTATION

The first case was of a 55‐year‐old, previously healthy, male patient. He presented with vague abdominal pain and discomfort after meals. He had a history of heavy alcohol consumption and smoking. His symptoms began 4 months before admission. Initially, he underwent an upper GI endoscopy that revealed an *H. pylori*‐positive gastritis. Thus, he was treated with proton pump inhibitors (PPIs) and antibiotic administration. Although his follow‐up endoscopy showed remission of the initial findings, the symptoms remained. He was afterward referred to our department. His physical examination revealed no particular findings apart from mild pain during the left upper abdominal quadrant palpation.

**Figure 1:**
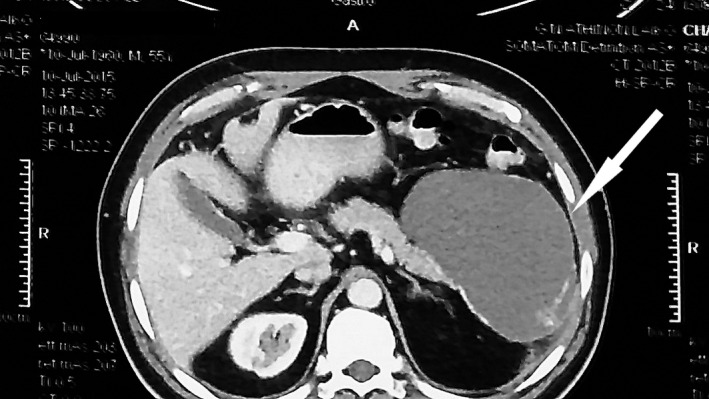
Computed tomography revealing a cystic mass at the tail of the pancreas

Patient's laboratory evaluation revealed elevated serum γ‐GT: 135 U/L, LDH: 250 U/L, and glucose: 165 mg/dL. Also, CA 19.9 was slightly elevated at 45 U/L. The rest of the laboratory values were within normal values. His upper abdominal ultrasound showed a mass located at the tail of the pancreas measuring 10 cm, and the computed tomography scan that followed revealed a cystic mass at the tail of the pancreas, with a max diameter of 12 cm (Figure 1). Following these findings, an abdominal magnetic resonance imaging scan confirmed the previous studies, with a well‐defined solid cystic lesion, unable to further specify character of the tumors. Also, the patient underwent endoscopic ultrasound (EUS) that showed a large solid, heterogeneous well‐circumscribed pancreatic cystic mass, but failed to aspirate content for cytology examination. As the EUS‐FNA performed could not exclude a potentially malignant cystic lesion, the suspicion of a cystic tumor of the tail of the pancreas leads the multidisciplinary team to suggest surgical exploration. Laparotomy revealed a cystic mass of the tail of the pancreas measuring 12 cm. In addition to this, the splenic vein was occluded by the mass prior to its confluence with the superior mesenteric vein. As a result, distal pancreatectomy and splenectomy were performed.

**Figure 2:**
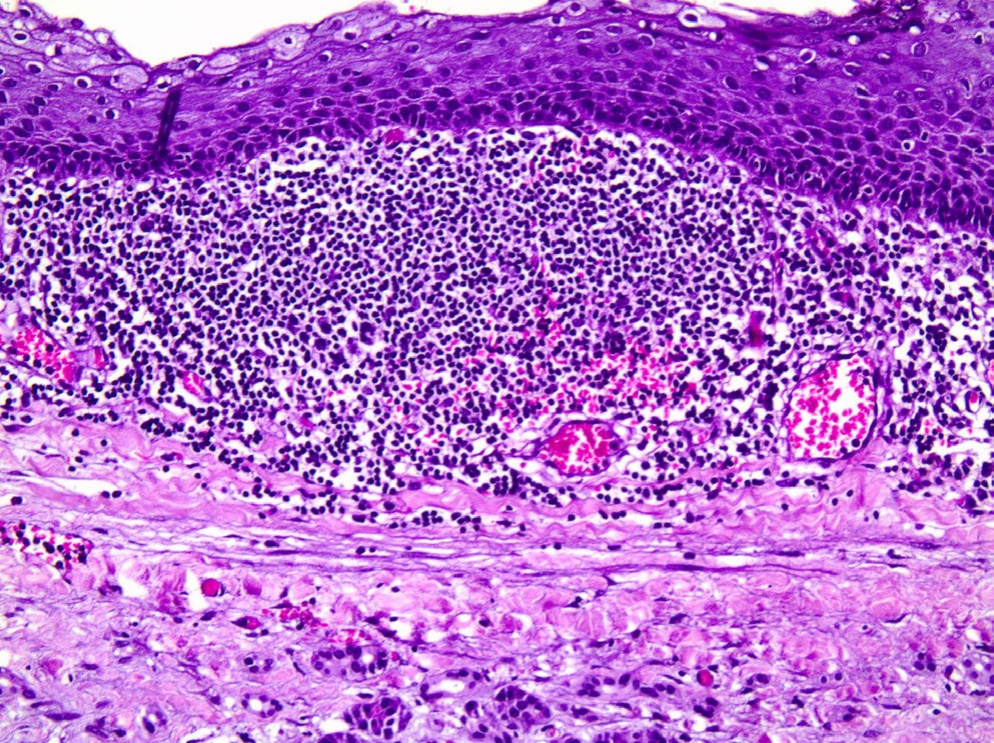
Lymphoepithelial cyst of pancreas consisted of squamous epithelium in close association with lymphoid tissue (H&E ×200)

Pathology report revealed a multiloculated cyst with cystic walls lined by keratinizing stratified squamous epithelium. Beneath the squamous lining epithelium, lymphoid tissue, plasma cells, and germinal centers were found, as well as multiple lymphoid follicles, without signs of atypia (Figure 2). Lymphoepithelial cyst of the pancreas was the conclusive diagnosis. Furthermore, submersions of the epithelium were identified to compose smaller cysts filled with mature keratinized material and surrounded by lymphoid tissue, along with lipoid tissue and lipoid‐necrotic cysts filled with macrophages and Langerhans giant cells. The cyst adhered to the pancreatic tail, where dense fibrous reaction with ipsilateral sclerosis and atrophy of the pancreatic parenchyma was present. Postoperative course was uneventful, and he was discharged on the 15th postoperative day. On his 6‐month follow‐up, he was relieved from his symptoms and no sign of recurrence was observed at his abdomen CT scan.

**Figure 3:**
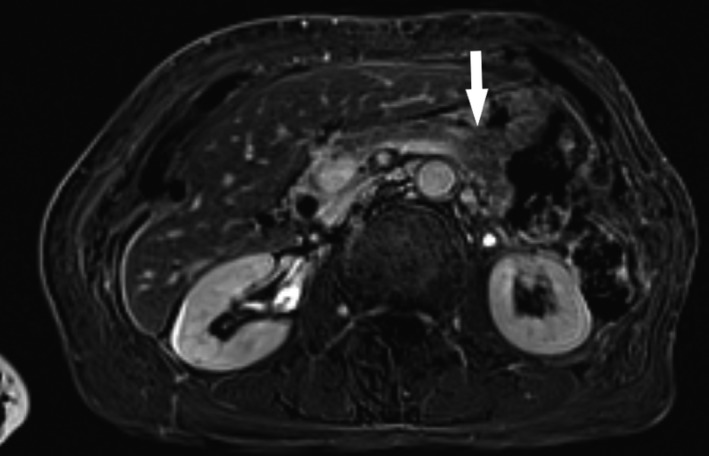
MRI revealing a 2.5‐cm pancreatic LEC at the body of the pancreas

The second case concerned a 67‐year‐old female patient, who presented with an incidental mass of the pancreas. She had undergone right hemicolectomy and segment VIII liver metastasectomy for colon adenocarcinoma 2 years before. During the follow‐up period, an incidental cystic lesion on the body of the pancreas was discovered on MRI and CT scans. The blood tests and serum tumor markers were within normal range. The mass was a unilocular cyst on the body of the pancreas, sizing 2.5 × 2 cm (Figure 3). In the wake of these findings, the patient was planned for EUS in order to exclude a potentially malignant cyst. EUS revealed a solid, heterogeneous, well‐circumscribed cystic mass measuring 2.3 × 2.2 cm on the body of the pancreas. EUS‐FNAB performed at that time revealed multiple follicles of keratinized material stratified with squamous epithelium along with lymphoid tissue, without signs of atypia. The diagnosis was compatible with pancreatic LEC. Due to the benign and asymptomatic nature of the lesion, and its incidental finding, the patient was managed conservatively despite patient's history of malignancy. Close follow‐up monitoring of the patient with regular laboratory and imaging studies was suggested with the possibility of lesion's progression, early detection, and surgical removal. Follow‐up laboratory examinations and CT/MRI scan were suggested every 3‐6 months for the first year. For the 2‐year follow‐up time, the patient remains asymptomatic free and there is no progression of the disease.

## 3 DISCUSSION

Cystic neoplasms and other cystic lesions of the pancreas, many of which can cause cystic dilatations of the main pancreatic duct and/or its branches, are collectively referred to as cystic lesions of the pancreas on cross‐sectional imaging. The incidence of these lesions increases with age, and it has been reported that up to one‐quarter of the elderly population harbor such lesions on autopsy reports. The increasing use of computer tomography (CT) and magnetic resonance (MRI) imaging studies of the abdomen during the last decades has resulted in an exponential increase in the frequency of discovery of such lesions even in asymptomatic patients.^8^ Some of these lesions can be clearly benign on diagnosis, whereas others can be malignant or have malignant potential.

Differential diagnosis of pancreatic cystic lesions is difficult, and physicians should be aware of the different types of cystic lesions and their characteristics before treatment selection. Pancreatic pseudocysts which are considered to be the most common nonneoplastic cysts of the pancreas do not represent true cystic lesions since their wall is not characterized by epithelial lining. Congenital cysts are rare and include those associated with genetic diseases such as autosomal dominant polycystic disease, cystic fibrosis, and von Hippel‐Lindau (VHL) disease. 90% of cystic neoplasms of the pancreas are represented by three types of lesions: Serous cystic neoplasms (SCNs) of the pancreas which due to the rare progression to malignancy can be managed conservatively provided that there are a definite diagnosis and absence of symptoms.^9^ Mucinous cystic neoplasms (MCNs) are premalignant in nature, can be invasive on diagnosis, and are best treated by resection in patients with acceptable operative risks. Furthermore, intraductal papillary mucinous neoplasms (IPMNs) represent cystic lesions with malignant potential and are considered amenable for surgical resection whenever the risk of malignant transformation exerts the risks of surgical management.^10^ Finally, solid pseudopapillary neoplasms with cystic component usually occur in young female patients. Resection of the primary tumor bears excellent survival results.

Lymphoepithelial cysts (LECs) are rare benign lesions of the pancreas lined with stratified squamous epithelium and surrounded by mature lymphocytes. LECs are a rare entity, and their history is better described and researched in the maxillofacial region. They are also characterized as branchial cysts since they are believed to arise from embryologic remnants of branchial cleft origin in the neck.^4^, ^5^ A case of LEC of the pancreas was first described by Luchtrach H and Schriefers K in 1985, and to the best of our knowledge, approximately 200 have been described in the English literature since then.^4^, ^5^

Due to their rare nature, lymphoepithelial pancreatic cysts are poorly characterized lesions, and their pathophysiology is not extensively studied. Different theories suggest that the formation of the cysts arises from squamous metaplasia of the pancreatic ducts or derivation from epithelial remnants in lymph nodes or possible displacement of branchial cysts during the pancreatic embryogenesis.^4^, ^5^

The patients are usually male (80%) with a mean age of 55 years on presentation. They can be asymptomatic and have the lesion discovered incidentally during imaging studies performed for other pathologies (43%) or can present with a variety of symptoms with vague abdominal pain (48%) being the most paramount, whereas back pain, impaired general condition, jaundice, and various gastrointestinal and respiratory complaints are rare.^5^, ^7^, ^11^

Laboratory examinations are usually equivocal showing transaminasemia, cholestasis, or amylasemia in few cases (8%). There is a trend toward a rise of serum CA 19‐9 in half of the cases (50%) and a rise in CEA in 6% of the cases, whereas the rest of serum tumor markers remain in normal range.^8^, ^12^ They can be located in the head (25%), the body (32%), or the tail (38%) of the pancreas; they vary in size and can be unilocular or multilocular.^8^

On imaging studies, LECs are often round with a well‐defined wall. The wall is sharply demarcated from the pancreas and surrounding adipose tissue. The main radiographic finding that may help distinguish LECs from other pancreatic cystic lesions is that they are sharply demarcated from the pancreatic tissue. The cyst content may display a “caseous” appearance characteristic of keratinaceous debris or may be clear and serous.^13^ Although the most commonly performed imaging study on admission remains the upper abdominal ultrasound, results are usually equivocal and thus are followed by more comprehensive studies such as abdominal CT scan and/or MRI studies. On CT studies, LECs are variably described as low attenuation cystic lesions harboring small solid component, showing papillary projections, wall calcification, and thin wall enhancement. In the majority of cases, LEC presents as either a multicystic mass or cyst with thin septations.^2^ On MRI, the cystic nature of the lesion can be clearly identified by its T1‐weighted hypointensity and T2‐weighted hyperintensity. However, cholesterol clefts in the keratin contents of the cyst may raise and suppress the signal intensities on T1‐ and T2‐weighted images, respectively. Fat saturation and chemical shift imaging techniques could be helpful to identify their fatty components.^14^

Cytological diagnosis of LEC through the use of EUS‐FNA is possible. EUS may reveal the cystic lesion on the pancreas raising the suspicion of a LEC diagnosis, while EUS‐FNA may set the diagnosis. On EUS examination, LECs appear solid, heterogeneous, and well‐circumscribed peripancreatic lesions. Cytological presence of squamous material and lymphocytes is diagnostic of LEC.^15^ The aspirate may be white or light brown that makes the differential diagnosis from a cystic neoplasm difficult. Neoplastic from benign cysts can be distinguished through cyst fluid analysis in which a cyst fluid CEA level > 200 ng/mL is strongly supportive of a diagnosis of MCN. However, LECs may also express elevated levels of CEA and Ca 19‐9, making the cyst fluid analysis an unreliable tool for the differentiation of LECs and malignant cystic lesions.^6^, ^12^, ^16^

Histological examination reveals a lined stratified squamous epithelium with an adjacent subepithelial layer of lymphoid tissue containing lymphoid follicles. Occasionally, mucinous cells have been present. Pathological analysis remains the gold standard for the diagnosis of pancreatic LECs.^12^

Surgery is still indicated in symptomatic patients or when malignancy cannot be excluded, as in our first patient. Pancreaticoduodenectomy and distal pancreatectomy are surgical options in large cysts locating in the head or tail of the pancreas. In smaller, well‐delineated lesions, cyst enucleation and drainage may be used if other macrocystic neoplasms have been excluded. Conservative management may be indicated in asymptomatic patients where a potential of malignancy has been excluded, as in our second patient, where “watchful wait” strategy may effectively avoid an unnecessary surgical resection.^12^

In conclusion, LECs of the pancreas are extremely rare entities. Although benign in nature, the differential diagnosis from other cystic pancreatic lesions with malignant potential is extremely difficult. Physicians should be aware of their existence, and high suspicion is crucial to exclude other malignant cystic pancreatic lesions. Both surgical resection and conservative management are effective strategies in managing LECs, and every patient should be approached individually for the most appropriate management.

## CONFLICT OF INTEREST

The authors declare no conflict of interest.

## AUTHOR CONTRIBUTIONS

All authors contributed equally to the manuscript.

## CONSENT

Written informed consent has been obtained from the patients.
